# A Method of Exactly Locating Gilmore’s Attachments

**Published:** 1915-01

**Authors:** 


### A METHOD OF EXACTLY LOCATING GILMORE'S ATTACHMENTS.

It may be of interest to users of Dr. Gilmore's attachments for plate and bridge work which are made by the Detroit Dental Mfg. Co., to read how Dr. Samuel P. Grant of Danville, Kentucky, makes sure of obtaining absolutely correct position and angle of the Gilmore attachments on the vulcanite plate. The abutments are finished with the pin and top piece of 14 gauge clasp round wire and put into position in the mouth and the Gilmore attachments placed on them in just the exact position in which they are to be when holding in the vulcanite. The impression is then taken in plaster paris with the attachments in this position. After the impression is removed from the mouth, the attachment clasps are detached from the bars in the mouth and are then placed in the imprints they made in the impression, dummy pieces of 14 gauge aluminum wire are then adjusted in the same position in the clasp attachment as the gold wire attached to the abutments; the ends of the aluminum wire being bent at right angle to stand out of the impression. When the model is poured, these right angle ends afford a hold on the aluminum wire to hold it and the attached clasps in position. Then pour the model with the attachments and dummy wires in position as above described. On separation you have the model with the aluminum wires holding the Gilmore attachments securely in the exact position in which you placed them in the mouth. Wax up and proceed as usual in plate work. If the aluminum wires hold in the impression they make in the rubber after separation from the plaster, the right angle tip affords a hold to enable their easy removal leaving the rubber so formed to fit the abutments.

